# Comparison and Supervised Learning of Segmentation Methods Dedicated to Specular Microscope Images of Corneal Endothelium

**DOI:** 10.1155/2014/704791

**Published:** 2014-09-22

**Authors:** Yann Gavet, Jean-Charles Pinoli

**Affiliations:** LGF UMR CNRS 5307, École Nationale Supérieure des Mines de Saint-Etienne, 158 Cours Fauriel, 42023 Saint-Etienne Cedex 2, France

## Abstract

The cornea is the front of the eye. Its inner cell layer, called the endothelium, is important because it is closely related to the light transparency of the cornea. An in vivo observation of this layer is performed by using specular microscopy to evaluate the health of the cells: a high spatial density will result in a good transparency. Thus, the main criterion required by ophthalmologists is the cell density of the cornea endothelium, mainly obtained by an image segmentation process. Different methods can perform the image segmentation of these cells, and the three most performing methods are studied here. The question for the ophthalmologists is how to choose the best algorithm and to obtain the best possible results with it. This paper presents a methodology to compare these algorithms together. Moreover, by the way of geometric dissimilarity criteria, the algorithms are tuned up, and the best parameter values are thus proposed to the expert ophthalmologists.

## 1. Introduction

### 1.1. Human Eye and Cornea

The eye is the first sense organ responsible for human vision. The human eye functions like a camera to refract light and produce a focused image which stimulates neural responses transmitted to the brain vision centers. crystalline lens is made of compacted protein fibers and is anchored in place by muscles attached to the wall of the eyeball. Contraction of these muscles causes the lens to change its shape and curvature, thus improving the focusing power. Refracted light passes through the eye cavity and strikes the inner surface at the back, known as the retina. The retina contains the specialized nerve cells called rods and cones that detect the intensity and the frequency of the incoming light. Light stimulates the rods and cones, which creates neural impulses that are transmitted to the brain through a network of nerve cells bunched together to form the optic nerve that exits from the back of the eyeball and passes to the brain.

#### 1.1.1. The Human Cornea

The cornea is the transparent, spherical surface covering the front of the eye. It is a powerful refractive surface, providing about 2/3 of the eye's focusing power. Healthy cornea has no blood vessel, which accounts for its clarity. But it is rich in nerve endings and so it is extremely sensitive to pain. The tears and aqueous humor, a watery fluid circulating in the cavity behind it that contains glucose and several electrolytes, nourish the cornea. The cornea is a highly organized tissue consisting of cells and protein arranged in three main layers:

- epithelium: this is the outermost layer comprising about 10% of the total thickness. Along with the tear film that bathes the outer surface of the eye, it provides a protective function preventing the entry of foreign material into the eye;
- stroma: it makes up to 90% of the corneal thickness. It consists primarily of water (78%) and layered collagen fibers (16%) that give the cornea its strength, elasticity, and shape. It also contains cells scattered between the fibers that produce the stromal constituents. The lattice-like arrangement and uniform spacing of the collagen fibers are essential for corneal transparency;
- endothelium: this is the innermost layer facing the aqueous and consists of a single layer of hexagonal cells. It pumps water out of the cornea and hence plays a vital role in keeping it in a dehydrated state. Without this pumping action, the stroma would accumulate water and become hazy and finally opaque (corneal oedema) leading to loss of vision.

#### 1.1.2. Physiology of the Human Corneal Endothelium

The cornea must remain transparent to refract light properly and the corneal endothelium ensures the integrity and transparency of the cornea.

The corneal endothelium consists of a single layer of closely packed, flat, hexagonally shaped cells covering the back surface of the cornea. In the human cornea at birth, there are more than 4000 cells/mm^2^. With age, the number of endothelial cells gradually decreases, but because they cannot regenerate, neighboring cells spread out to fill the gap leading to an alteration of cell shape (pleomorphism) and size (polymegathism). The mean endothelial cell density (ECD) in adults is generally between 500 and 3500 cells/mm^2^. Cell density, as well as variation in size and shape, can be examined by specular microscopy in living human subjects. These methods permit early diagnosis of any damage of the corneal endothelium.

### 1.2. Principles of Specular Microscopy

Since the cornea is transparent, cornea cells can easily be observed in vivo with a specular microscope. This technology comes from the early 1980s. Those optical microscopes can acquire an image of the cells on a very little surface (0.08 mm^2^ compared to the endothelium surface of about 100 mm^2^; see Figure 5). The image is then analyzed by a computer software (embedded in the microscope) to provide both cell density and morphometry. The physical principle of this type of microscope is the specular reflection (i.e., the same reflection as for a mirror). As the light goes through the several different layers of the cornea, it is reflected at each interface [1, 2]. The deeper the layer is in the cornea, the darker it appears on the image. This explains the intensity variations within the images, which physically corresponds to the superposition of several different layers.

### 1.3. Quality Criteria of the Corneal Endothelium

It is necessary to evaluate the quality of the human corneal endothelium in several circumstances (for example, after accidents, surgery, or trauma). The main cause is corneal grafting.

The two criteria required for the evaluation are

- the endothelial cell density (ECD, in cells/mm^2^): there are several threshold values: for example, an ECD lower than 400 cells/mm^2^ does not enable maintaining the cornea transparency. An ECD lower than 1000 cells/mm^2^ is a contraindication for using intraocular lens implants.
- the morphometry of endothelial cells: their size regularity (called the polymegathism, i.e., the variation of areas of the cells) and their shape regularity (called the pleomorphism, i.e., the percentage of hexagon-like cells) induce a good quality of the cornea.

## 2. Three Image Segmentation Methods

Different methods exist to perform the segmentation of images of endothelial cells. Among those, three methods give the better results [3]. The present paper first recalls their related algorithms and will then compare their results with regard to several criteria.

The presented algorithms have a common structure. First, they filter the original image. Second, they aim to find some markers of the cells, and then they perform a morphological operation (a watershed; see [6]) to get closed contours for each cell. Notice that these three algorithms make important use of mathematical morphology operators (see, e.g., [7]).

### 2.1. Vincent and Masters' Method

This method has been proposed in [4]. It is based on the fact that cell borders' intensities are lower than the cell interiors' intensities and represent somehow a local maximum of intensity that is retrieved by a morphological operation called a *h*-maxima [7]. To avoid the problem of noise, a first filtering process is performed by the way of a morphological alternate sequential filter. It involves two parameters.

- *h* is a value for the *h*-maxima operation (an intensity) that gives the final markers of the cells.
- *o* is the order of the morphological alternate sequential filter.

The algorithm is summarized in Algorithm 1.

### 2.2. Angulo and Matou's Method

This method is more recent than the previous one [5]. The cell markers are detected by the mean of a distance map after some filtering process (performed by a morphological opening and a morphological top-hat). The difference with Vincent and Masters' algorithm is that a first segmentation of the contours is performed and then is improved by the use of the watershed [6].  Algorithm 2 details the algorithm. Five parameters are required. The three first ones, *s*_1_, *s*_2_, and *g*, are used for the filtering process. The last two ones, *t* and *h*, are used to get the markers that will be used by the watershed.

### 2.3. Gavet and Pinoli's Method

This is the most recent method proposed in [3]. The improvements to the other methods come from the fact that the first segmentation of the borders of the cells better performs due to the elimination of nonlinear parts of the skeleton (see details of Algorithms 2 and 3). The Gavet and Pinoli's method requires five parameters, and its algorithm is summarized in Algorithm 3. After a filtering process (parameter *o*), the contours are first roughly detected by the use of the openings by segments (parameters *t*_*s*_ and then *o*, *c*, and *e*). This operators intend to detect linear cell contours. Then, these contours are corrected by the mean of a distance map and a constrained watershed (parameter *s*), as proposed by the two previous methods.

### 2.4. Summary of the Control Parameters

Table 1 summaries the control parameters used by the three presented image segmentation methods. The main problem here is how to select the values of these parameters. The following section will try to answer this problem by using two comparison criteria (namely, the dissimilarity criteria *ϵ* and fom) and by using them on an image database to find the best parameter values.

## 3. Image Segmentation Evaluation

The evaluation of a segmentation quality is a common problem encountered when developing a segmentation method. Like the segmentation methods themselves, the image segmentation evaluation criteria can be classified into region-based or contour-based approaches, although they usually can be adapted from one class to the other. The segmentation processes of the corneal endothelium result in the contours of the cells, but the proposed comparison methods are also suitable for segmented regions.

This paper deals with supervised segmentation evaluation, that is, involving a criterion that compares the result of the segmentation process to a ground truth image (usually manually segmented by an expert of the application field). This is usually preferred to unsupervised evaluation (where some kind of intraregion homogeneity is involved), but the bias introduced by the expert does not have to be neglected (see Section 3.4).

### 3.1. Basic Notations

The following notations are first introduced: *I*_*R*_ and *I*_*X*_ represent two binary images that correspond to a reference segmentation method *R* and the evaluated segmentation method *X*, respectively. Both *I*_*R*_ and *I*_*X*_ are considered as sets of contours. In this paper, *R* and *X* may be employed for *I*_*R*_ and *I*_*X*_ in order to alleviate the notations and more deeply for emphasizing the geometrical problems. A point *p* ∈ *I*_*X*_ or *p* ∈ *I*_*R*_ means a point present in the related segmented binary image.

### 3.2. Classical Dissimilarity Criteria

This paper will not present an exhaustive view of supervised evaluation of segmentation criteria. The reader can have a look at [8] for a more complete presentation and a comparison.

The two detailed criteria have been chosen because they are tolerant towards spatial variations. One could also use other frequently used criteria proposed in the literature [9–11], like the Hausdorff distance, the Dice coefficient (also known as the Jaccard index), or classification criteria (specificity, sensibility). The main drawback of these criteria is that a small noise (like a misdetected point) implies a high comparison value. This is why people introduced a distance ponderation, like the *p*th point in the Hausdorff distance, or the following figure of merit.

The figure of merit [12, 13] is defined by

(1) $$\begin{array}{c} \text{fo}{\text{m}}_{R}\left(X\right)=1-\frac{1}{\max\left\{\#\left(M\right),\#\left(X\right)\right\}}\underset{p\in X}{\sum }\frac{1}{1+d^{2}\left(p,R\right)}, \end{array}$$

where *d*(*p*, *R*) is the Euclidean distance from the pixel *p* ∈ *X* to the closest pixel of *R*, and # is the number of pixels of the considered segmentation result *R* or result *X* (which are nonempty images, at least for *R*).

#### 3.2.1. Partitioning

An image segmentation process refers to the action of partitioning the spatial domain of an image into adjacent regions, each of them preserving a certain homogeneity following a given criterion. Thus, a computer program is able to answer the following binary question: is this pixel inside the region of interest or not?

To formalize this mathematically, let *S* be a binary image resulting from a segmentation process, defined by the number of regions (number of labels *L*, *K* = [1; *L*]) that partitions the spatial domain *D* and by the set *R* of adjacent regions *R*_*i*_ that fulfill *D*:

(2) $$\begin{array}{c} \forall \left(i,j\right)\in K,\;\;i\neq j,\;R_{i}\cap R_{j}=\emptyset ,\\ R=⋃R_{i},\;i\in K. \end{array}$$

This paper deals with the case where contours are detected and the segmentation result is a binary image; that is, *L* = 2, *K* = [1; 2] (label 1 stands for the background and label 2 for the detected contours).

### 3.3. The *ϵ* Dissimilarity Criterion

The *ϵ* dissimilarity criterion is based on the symmetric difference Δ of sets, but this latter lacks some tolerance, which is introduced by the Minkowski addition.

#### 3.3.1. Symmetric Difference Set

First, let us recall that the symmetric difference set between two segmentations *R* and *X* (*R* ⊂ *D* and *X* ⊂ *D*), denoted by Δ(*R*, *X*), is defined by

(3) Δ(R,X)=(R∪X)∖(R∩X)=(R∖X)∪(X∖R).

#### 3.3.2. Minkowski Addition

The Minkowski addition [14] defines an algebraic operation between sets in the *n*-dimensional Euclidean space *R* ^*n*^. It will be used to spatially “enlarge” the segmentations *X* or *R* to be compared in order to be less sensitive to small spatial variations or noises.

If *X* is a set (segmentation result) and *B* is another set (generally the unit ball), the Minkowski sum of *X* and *B*, denoted by *X* ⊕ *B*, is then defined by

(4) $$\begin{array}{c} X⊕B=\left\{x+b\;|\;x\in X,b\in B\right\}\\ X⊕B=\underset{b\in B}{⋃}\left\{x+b\;|\;x\in X\right\}, \end{array}$$

where ⊕ is the Minkowski addition symbol. In the field of mathematical morphology [7], it is equivalent to the dilation, and *B* is called a structuring element (for example, the unit ball).

#### 3.3.3. Definition

In [15], each pixel in the segmentation result is attributed a distance value to the reference segmentation, and a histogram of these distances is thus computed. Then, a statistical analysis of this histogram is performed. In the same spirit, we propose a dissimilarity criterion that is tolerant towards small spatial variations. The *ϵ* dissimilarity criterion with the tolerance *ρ* applied to segmented images is defined in the case of discrete images (*R* is the reference segmentation result and *X* is the considered segmented image) by [8]

(5) $$\begin{array}{c} \epsilon_{R}^{\rho }\left(X\right)=\frac{\#\left\{\left(X\setminus \left(R⊕\rho N\right)\right)\cup \left(R\setminus \left(X⊕\rho N\right)\right)\right\}}{\#\left\{R⊕\rho N\right\}} \end{array}$$

with *N* being the structuring element of radius 1 (typically the unit ball) and # designating the number of pixels in the set (# is the cardinal operator, counting the number of nonzero valued pixels in the set *X* or set *R*). Practically, *ρ* is the radius of the ball used to dilate the binary images, thus forming a tolerance tube around the original set *X* or set *R*. This paper will propose a way of selecting the right value for *ρ*.

The main properties of *ϵ* are

- *ϵ*_*R*_^*ρ*^(*R*) = 0, which means that when *R* is compared to itself, the numerical result is 0;
- $\epsilon_{R}^{\rho }(R)\underset{\rho \to \infty }{\to }0$, which means that if the tolerance increases, the numerical value tends to 0;
- *ρ* is the tolerance value; thus, *ϵ* is tolerant towards small spatial variations (like translations, rotations, and over- and undersegmentations).

#### 3.3.4. Discussion about the Notion of Metric

The usual concept to compare mathematical objects is the metric notion, defined by four axioms (identity, separation, symmetry, and triangle inequality; see [16]). If a metric has important mathematical properties, it has been proved that the human visual system does not follow them [17, 18]. For example, the human visual system does not always consider two distinct objects as different (the separation property is thus not verified). This is also true for the triangle inequality and the symmetry property [8]. It is important to notice that *ϵ* is not a metric: separation, symmetry, and triangle inequality are not verified. This is why it is called a dissimilarity criterion.

### 3.4. Bias in Experts Manual Segmentation: Choice of the Tolerance Value

The problem of the experts reference segmentation is crucial because subject to variations between experts and sometimes also for one expert. To deal with this problem, some articles use an average result, like [19]. Some others do not take these into account and use only one reference segmentation as an absolute truth. The proposed *ϵ* dissimilarity criterion deals with this problem by the choice of the tolerance parameter *ρ*. The *ρ* value will in fact reflect the precision of the manual segmentation of the expert.

**Figure 1:**
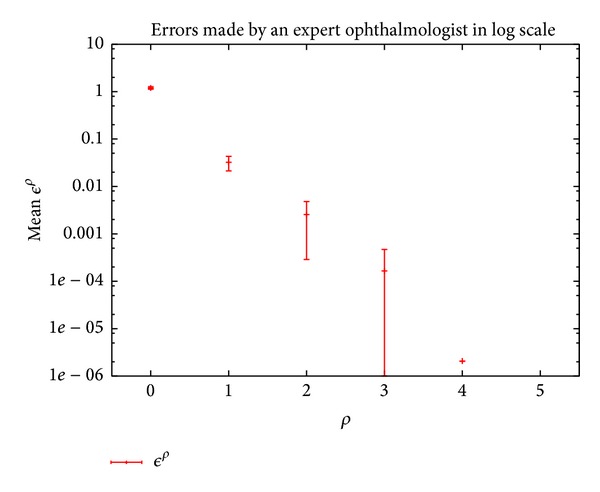
Method for fixing the tolerance parameter. In this example, *ρ* is in pixels, and there is a strong gap between no tolerance (*ρ* = 0) and a tolerance of one pixel (*ρ* = 1). The scale is logarithmic.

For one original gray-tone image, the experts have manually drawn their segmented image several times, and the *ϵ* dissimilarity criterion has been used to compare every manually segmented image to the others. The mean value of the *ϵ* dissimilarity criterion is represented in Figure 1. The reader can consider that an expert will always (try to) draw the contours at the same location within a certain spatial tolerance (i.e., within the tolerance tube), depending on the image size and the precision of the drawing tool. In the Figure 1, if an error is fixed at a maximum of *ϵ* = 0.05, the application should then use a tolerance value of *ρ* = 2.

Thus, the *ϵ* dissimilarity criterion is able to deal with the bias in the experts reference segmentation as well with the noises present in the segmentation results themselves. The next section will focus on cornea endothelium images and their segmentations.

## 4. Image Segmentation Method Tuning

The different segmentation algorithms presented in the previous sections require to setup the values of the so-called control parameters. The choice of the control parameter values for a specific application issue is generally not trivial, especially for nonimage analysis experts. This section explains the generic way of selecting the best parameters in average for the considered three image segmentation methods.

### 4.1. Method

#### 4.1.1. Definitions

Let *p* be the control parameters set of a given algorithm *A*. For example, for Algorithm 1, *p* = {*s*, *h*}. Then, *A*_*p*_(*I*) is the result of the segmentation process by algorithm *A* with the parameter set *p* on the input image *I*.

Let *C* denote the criterion used to compare the segmentation results with the reference. In this paper, *C* will be either the dissimilarity criterion *ϵ* or criterion fom.

Let *Q* be an evaluation function of the quality of the segmentation, depending on the considered criterion *C*, defined as follows:

(6) $$\begin{array}{c} Q_{C}\left(A_{p},I\right)=C\left(A_{p}\left(I\right),R\right), \end{array}$$

where *R* is the reference segmentation of the image *I*. To simplify the notations, *R* will be used instead of *R*(*I*).

#### 4.1.2. Best Parameter Set

In the following, we consider an image database of *N* gray-tone images, each being associated with a reference segmented image. What we are looking for is the best parameter set, that is, the parameter set that will result in the best segmented images considering the reference *R* and a specific comparison criterion (among the two considered criteria, fom and *ϵ*).

Let $\hat{p}$ be the best parameter set regarding the mean of all quality values on the *N* gray-tone images of the database, yielding to

(7) p^(A,C)=arg min⁡p{1N∑IQC(Ap,I)}=arg min⁡p{meanI QC(Ap,I)}.

Let $\hat{Q}$ be the minimal mean value of *Q* on the *N* images of the database, yielding

(8) Q^(A,C)=min⁡p{1N∑IQC(Ap,I)}=meanI QC(Ap^,I).

This way of finding the best parameter set is also called leave-one-out cross validation.

#### 4.1.3. Trimmed Mean

Some noise may be present in the computed values (mainly because of a too poor image quality). To be more tolerant towards these perturbations, the trimmed mean (sometimes called truncated mean) is also employed: in the addressed application issue, given parts of the sample are discarded at the high end.

If *k* ∈ [0; 0.5] is the percentage of discarded values, then

(9) $$\begin{array}{c} {\hat{p}}_{k}\left(A,C\right)=\underset{p}{\arg\;\min}\left\{\underset{I,k}{\text{Tmean}}\;Q_{C}\left(A_{p},I\right)\right\},\\ {\hat{Q}}_{k}\left(A,C\right)=\underset{p}{\min}\left\{\underset{I,k}{\text{Tmean}}\;Q_{C}\left(A_{p},I\right)\right\}. \end{array}$$

Notice that the trimmed mean corresponds to the classical mean for *k* = 0; namely,

(10) $$\begin{array}{c} {\hat{p}}_{0}=\hat{p}\\ {\hat{Q}}_{0}=\hat{Q}. \end{array}$$

#### 4.1.4. Median

The median of *Q*, denoted by med *Q*, is also a classical way to avoid noise perturbations in such measurements, yielding to

(11) $$\begin{array}{c} \tilde{p}\left(A,C\right)=\underset{p}{\arg\;\min}\left\{\underset{I}{\text{med}}\;Q\left(A_{p},I\right)\right\}\\ \tilde{Q}\left(A,C\right)=\underset{p}{\min}\left\{\underset{I}{\text{med}}\;Q\left(A_{p},I\right)\right\}. \end{array}$$

#### 4.1.5. Projection

In order to observe the influence of one control parameter in the segmentation results, it is interesting to fix every control parameter but the considered one, and see if there is an impact on the quality of the segmentation. Let *P* be a parameter of the set *p*. Let ${\hat{Q}}_{k}^{P}$ be the (trimmed) mean evolution of *Q* when the parameter *P* is varying and the other parameters are fixed at values of ${\hat{p}}_{k}$. The parameters are chosen among those defined in Table 1.

#### 4.1.6. *K*-Fold Cross Validation

The *K*-fold cross-validation consists in validating the learning process by splitting the database into *K*-folds, using *K* − 1-folds as the learning database and the last one as the test database [20]. This is repeated *K* times such that each fold will be used as the test database. The result is a discrepancy value that reflects the pertinence of the learning. It will be noticed *CV* in the different result tables, which is the mean result value over each test partition.

The results will be presented in Tables 5, 6, and 7 in detail. For each method and for each partition, the learned parameter values are presented as well as the corresponding result of the criterion value for the test partition. Let *Q*_*i*_^*CV*^ be the value of the criterion for the partition *i*, and *Q* ^*CV*^ = mean_*i*_{*Q*_*i*_^*CV*^}.

For comparison purposes, we also provide the best criterion value that could have been obtained on the test partition, denoted by ${\tilde{Q}}_{i}^{CV}$ (for partition *i*) and by ${\tilde{Q}}^{CV}$ for the mean on all partitions. The value of ${\tilde{Q}}^{CV}$ should be only a little smaller than *Q* ^*CV*^.

### 4.2. Quantitative Comparison Results

This section presents the results for the three aforementioned image segmentation methods.

An image database of *N* = 30 gray-tone images of the human corneal endothelium acquired with a specular microscope is employed to evaluate the segmentation processes realized by the different algorithms. This image database (see Figure 5) contains gray-tone images and also the related experts' segmented images (manually performed).

#### 4.2.1. Vincent and Masters' Method

The summary of the optimal control parameters values is presented in Table 2.

**Figure 2:**
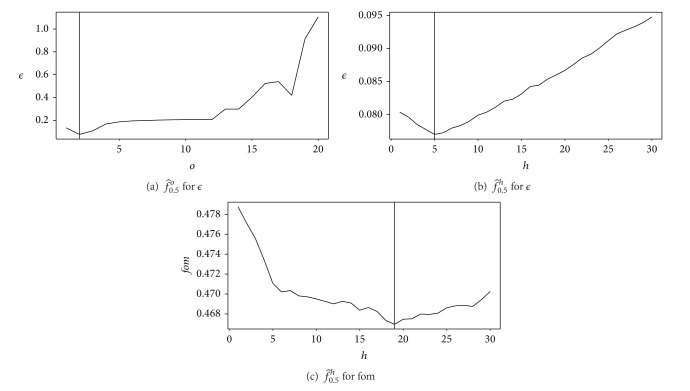
Projection on the optimal values of the control parameters *o* and *h* for the method of Vincent and Masters. See Table 2 for numerical values.

It appears that *ϵ* and fom do not provide the same results for the optimal value of parameter *h*. One shall notice that the value of ${\hat{Q}}_{0.5}^{h}$ does not vary a lot for both *ϵ* and fom criteria (see Figures 2(b) and 2(c)). This means that the choice of *h* appears as not crucial.

#### 4.2.2. Angulo and Matou's Method

**Figure 3:**
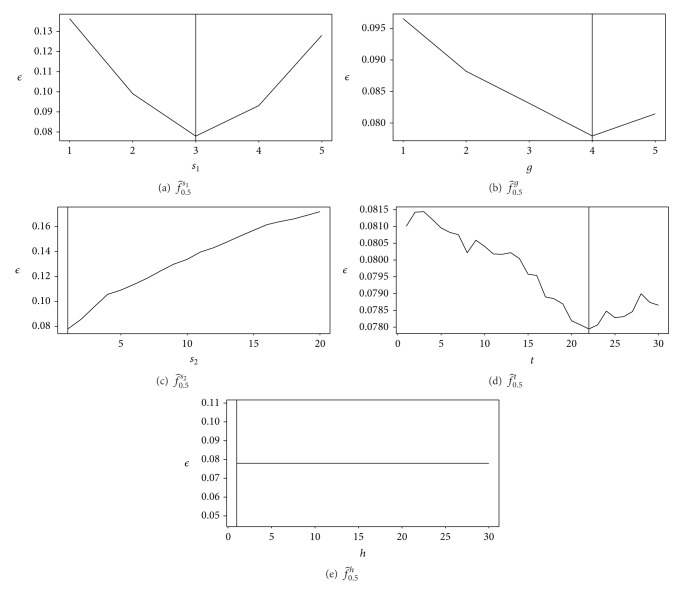
Projection on the optimal values of the control parameters for the method of Angulo and Matou. See Table 3 for numerical values.

The results are presented in Table 3. Both *ϵ* and fom give the same results. In addition, the projections (Figures 3(e) and 3(c)) show that *s*_2_ and *h* are useless. Thus, the top-hat transform can be avoided, and the *h*-maxima operation can be replaced by the computation of the maxima of the distance map.

#### 4.2.3. Gavet and Pinoli's Method

The summary of the optimal control parameter values is presented in Table 4.

**Figure 4:**
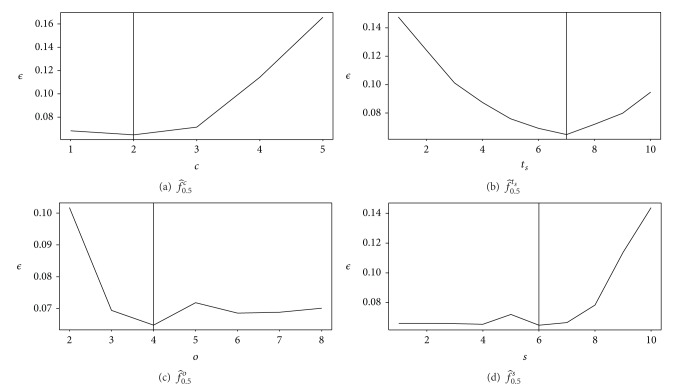
Projection on the optimal values of the control parameters for the method of Gavet and Pinoli. See Table 4 for numerical values.

**Figure 5:**
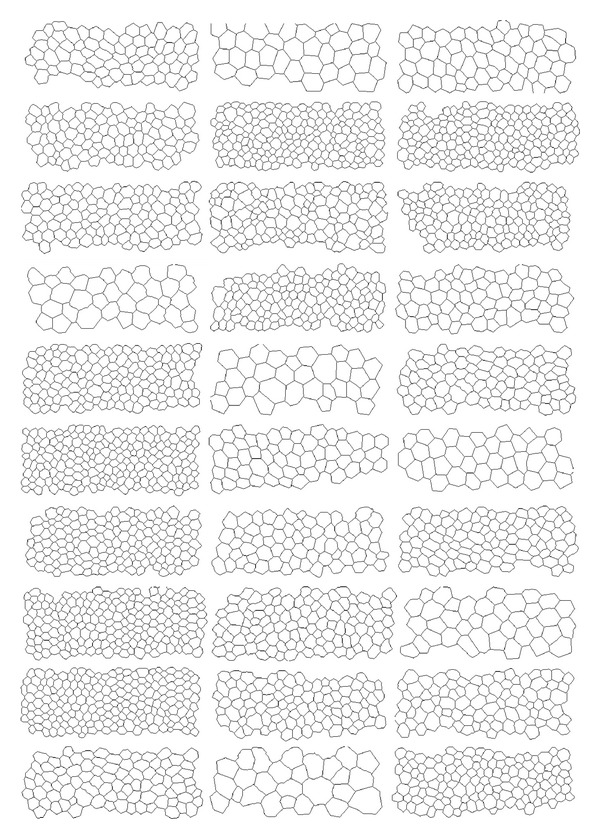
Table of the 30 reference segmented images of the database. They have been manually drawn by an expert ophthalmologist from a human corneal endothelium image database (see Figure 6). These images come from [8].

For both fom and *ϵ* criteria, the optimal control parameter values are identical. The filtering parameter *o* used in the alternate sequential filter must be higher than 4. This parameter is linked to the length of an edge of a cell, and, thus, some corneal endothelium with big cells would get a better result with a higher value of *o* (this explains the almost constant values that can be observed in Figure 4(c)).

The control parameter *s* used for filtering markers must be chosen with a low value (less than 7). In this case, the effect is not really noticeable.

The control parameter *t*_*s*_ is strictly related to the length of the cells edges (its purpose is to retrieve the linear parts of the detected borders and thus eliminate the noise).

### 4.3. Comments

According to the numerical values, the method of Gavet and Pinoli outperforms the methods from Vincent and Masters, and Angulo and Matou (see the values of *Q* ^*CV*^, $\hat{Q}$, ${\hat{Q}}_{0.5}$, or $\tilde{Q}$). Clearly, the superiority of this method comes from the extraction of the linear parts of the cell borders.

The *K*-fold cross-validation (see Tables 5, 6, 7, 8, 9, and 10) shows that the learning is not far from the optimal value, in terms of *ϵ* or fom, as well as in terms of optimal parameter values. This can be concluded from comparing the mean *ϵ* or fom value from the test partitions (*Q* ^*CV*^) to the best possible value (${\tilde{Q}}^{CV}$): there is only a small difference between these values.

Moreover, the optimal parameter values obtained for the different training partition do not vary a lot and are really similar to those proposed in Tables 2, 3, and 4.

## 5. Conclusion and Perspectives

In this paper, three segmentation methods suitable for binarizing the optical specular microscopy gray-tone images of human corneal endotheliums have been presented. These methods involve different control parameters. This is always a hard problem for the user because he has no time to manually tune up his computer softwares (and especially his image segmentation softwares). Two dissimilarity criteria have been employed (*ϵ* dissimilarity criterion [8] and Pratt's figure of merit fom [12]) to tune up the segmentation algorithms in regard to the expert manual segmentation. As a result, this paper proposes the optimal control parameter values to use for these images. It also proposes to avoid some operations since their parameters do not really influence the segmentation results. More generally, this paper highlights the relevance of the *ϵ* dissimilarity criterion to a spatial tolerance, suitable to handle the problem of the bias in the reference segmentation. This *ϵ* dissimilarity criterion is adapted to compare binary contour images as well as binary sets, for 2D or even 3D images. In a near future, we expect to report such a criterion to compare gray-tone images.

## Figures and Tables

**Figure 6:**
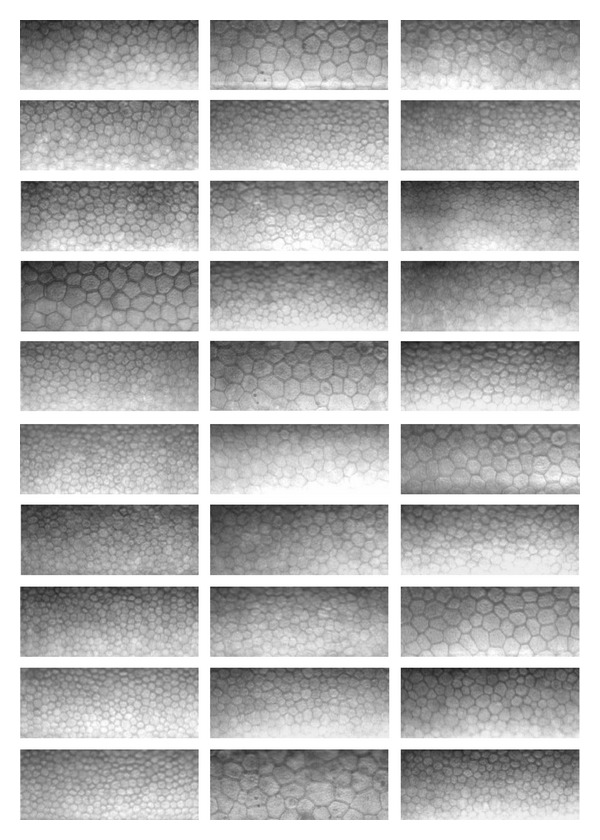
Table of the 30 specular microscopy images of corneal endotheliums of the database. They are segmented by the proposed method and by an ophthalmologist (see Figure 5). These images come from [8].

**Algorithm 1:**
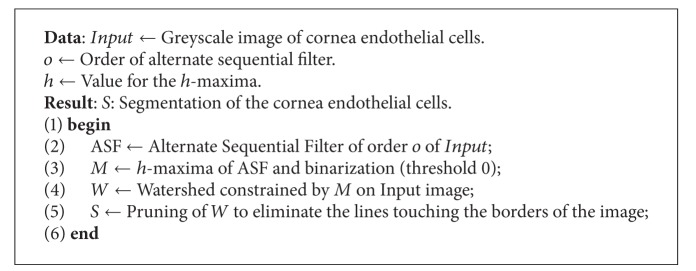
Vincent and Masters's algorithm for detecting the human corneal endothelium cells [4].

**Algorithm 2:**
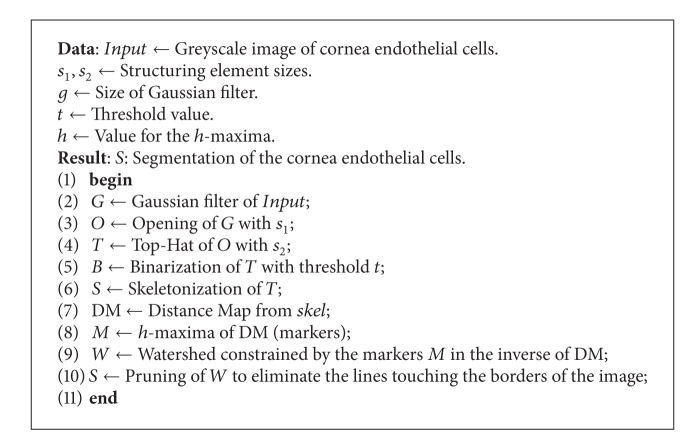
Angulo and Matou's algorithm for detecting the cells [5].

**Algorithm 3:**
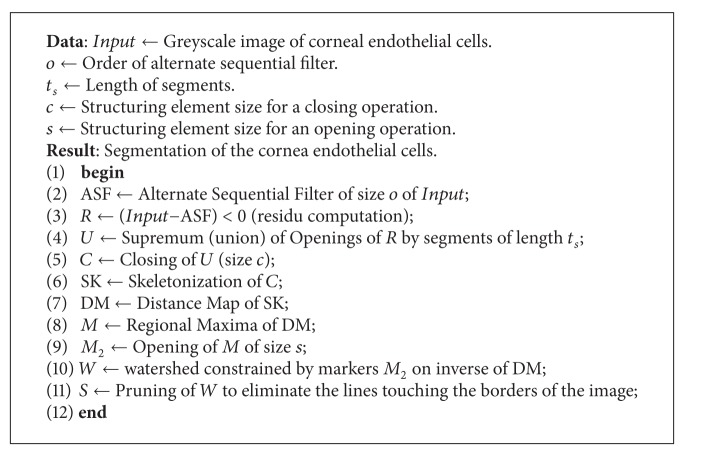
Gavet and Pinoli's algorithm for detecting the cornea endothelial cells.

**Table 1:** Summary of the control parameters of the three presented image segmentation algorithms.

Method	Parameters *p*	Description
Vincent and Masters Algorithm 1	{*o*, *h*}	*o*: order of filter
		*h*: *h*-maxima

Angulo and Matou Algorithm 2	{*s*_1_, *g*, *s*_2_, *t*, *h*}	*s*_1_: opening
		*g*: Gaussian filter
		*s*_2_: top-hat
		*t*: threshold
		*h*: *h*-maxima

Gavet and Pinoli Algorithm 3	{*c*, *t*_*s*_, *o*, *s*}	*c*: closing
		*t*_*s*_: length of segments
		*o*: order of filter
		*s*: opening

**Table 2:** Results for Algorithm 1, method of Vincent and Masters, where the control parameters are *p* = {*o*, *h*}. See Figure 2 for illustrations.

Criterion	Optimal parameters		Trimmed mean (*k* = 0.5)		Median		*Q* ^*CV*^
	$\hat{p}$	$\hat{Q}$	${\hat{p}}_{0.5}$	${\hat{Q}}_{0.5}$	$\tilde{p}$	$\tilde{Q}$	
*ϵ*	{2,12}	0.11	{2,5}	0.08	{2,5}	0.10	0.115
fom	{2,19}	0.52	{2,19}	0.47	{2,23}	0.50	0.520

**Table 3:** Results for Algorithm 2, method of Angulo and Matou, where the control parameters are *p* = {*s*_1_, *g*, *s*_2_, *t*, *h*}. See Figure 3 for illustrations.

Criterion	Optimal parameters		Trimmed mean (*k* = 0.5)		Median		*Q* ^*CV*^
	$\hat{p}$	$\hat{Q}$	${\hat{p}}_{0.5}$	${\hat{Q}}_{0.5}$	$\tilde{p}$	$\tilde{Q}$	
*ϵ*	{4,4, 1,28,1}	0.15	{3,4, 1,22,1}	0.08	{4,3, 1,16,1}	0.11	0.150
fom	{3,4, 1,21,1}	0.54	{3,4, 1,21,1}	0.46	{3,4, 1,8, 1}	0.51	0.525

**Table 4:** Results for Algorithm 3, method of Gavet and Pinoli, where the control parameters are *p* = {*c*, *t*_*s*_, *o*, *s*}. See Figure 4 for illustrations.

Criterion	Optimal parameters		Trimmed mean (*k* = 0.5)		Median		*Q* ^*CV*^
	$\hat{p}$	$\hat{Q}$	${\hat{p}}_{0.5}$	${\hat{Q}}_{0.5}$	$\tilde{p}$	$\tilde{Q}$	
*ϵ*	{2,7, 4,6}	0.10	{2,7, 4,6}	0.06	{2,7, 4,6}	0.08	0.099
fom	{2,7, 4,7}	0.50	{2,7, 4,7}	0.45	{2,7, 4,8}	0.49	0.506

**Table 5:** Cross-validation information for Algorithm 1, method of Vincent and Masters, with *ϵ* criterion.

Partition number	Optimal parameter values	*Q*_*i*_^*CV*^	${\tilde{Q}}_{i}^{\mathrm{CV}}$
1	{2,8}	0.146	0.118
2	{2,12}	0.099	0.096
3	{2,9}	0.110	0.109
4	{2,12}	0.117	0.115
5	{2,12}	0.104	0.104

Mean		*Q* ^*CV*^ = 0.115	${\tilde{Q}}^{\mathrm{CV}}=0.108$

**Table 6:** Cross-validation information for Algorithm 1, method of Vincent and Masters, with fom criterion.

Partition number	Optimal parameter values	*Q*_*i*_^*CV*^	${\tilde{Q}}_{i}^{\mathrm{CV}}$
1	{2,19}	0.512	0.512
2	{2,18}	0.524	0.510
3	{2,19}	0.490	0.488
4	{2,19}	0.533	0.533
5	{2,19}	0.540	0.540

Mean		*Q* ^*CV*^ = 0.520	${\tilde{Q}}^{\mathrm{CV}}=0.516$

**Table 7:** Cross-validation information for Algorithm 2, method of Angulo and Matou, with *ϵ* criterion.

Partition number	Optimal parameter values	*Q*_*i*_^*CV*^	${\tilde{Q}}_{i}^{\mathrm{CV}}$
1	{4,5, 1,25,1}	0.162	0.148
2	{4,4, 1,28,1}	0.157	0.155
3	{4,4, 1,28,1}	0.141	0.141
4	{4,4, 1,28,1}	0.109	0.109
5	{4,4, 1,30,1}	0.180	0.158

Mean		*Q* ^*CV*^ = 0.150	${\tilde{Q}}^{\mathrm{CV}}=0.142$

**Table 8:** Cross-validation information for Algorithm 2, method of Angulo and Matou, with fom criterion.

Partition number	Optimal parameter values	*Q*_*i*_^*CV*^	${\tilde{Q}}_{i}^{\mathrm{CV}}$
1	{3,4, 1,21,1}	0.510	0.510
2	{3,4, 1,21,1}	0.565	0.558
3	{3,4, 1,21,1}	0.624	0.598
4	{3,4, 1,21,1}	0.448	0.443
5	{3,4, 1,21,1}	0.479	0.479

Mean		*Q* ^*CV*^ = 0.525	${\tilde{Q}}^{\mathrm{CV}}=0.518$

**Table 9:** Cross-validation information for Algorithm 3, method of Gavet and Pinoli, with *ϵ* criterion.

Partition number	Optimal parameter values	*Q*_*i*_^*CV*^	${\tilde{Q}}_{i}^{\mathrm{CV}}$
1	{2,7, 4,6}	0.078	0.078
2	{2,7, 4,6}	0.100	0.099
3	{2,7, 4,5}	0.111	0.093
4	{2,7, 4,6}	0.109	0.101
5	{2,7, 4,7}	0.100	0.091

Mean		*Q* ^*CV*^ = 0.099	${\tilde{Q}}^{\mathrm{CV}}=0.092$

**Table 10:** Cross-validation information for Algorithm 3, method of Gavet and Pinoli, with fom criterion.

Partition number	Optimal parameter values	*Q*_*i*_^*CV*^	${\tilde{Q}}_{i}^{\mathrm{CV}}$
1	{2,7, 4,8}	0.506	0.502
2	{2,7, 4,7}	0.490	0.486
3	{2,7, 4,8}	0.486	0.480
4	{2,7, 4,7}	0.484	0.477
5	{2,7, 4,7}	0.562	0.548

Mean		*Q* ^*CV*^ = 0.506	${\tilde{Q}}^{\mathrm{CV}}=0.498$
